# Influenza A virus induced bacterial otitis media is independent of virus tropism for α2,6-linked sialic acid

**DOI:** 10.1186/1743-422X-10-128

**Published:** 2013-04-23

**Authors:** Kirsty R Short, Marrit N Habets, Jean Payne, Patrick C Reading, Dimitri A Diavatopoulos, Odilia L Wijburg

**Affiliations:** 1 Department of Microbiology and Immunology, The University of Melbourne, Melbourne, VIC, 3010, Australia; 2 Laboratory of Pediatric Infectious Diseases, Department of Pediatrics, Radboud University Medical Centre, Nijmegen, The Netherlands; 3 Australian Animal Health Laboratory, Geelong, VIC, 3220, Australia; 4 WHO Collaborating Centre for Reference and Research on Influenza, Victorian Infectious Diseases Reference Laboratory, Melbourne, VIC, 3051, Australia

**Keywords:** Otitis media, Viral-bacterial co-infection, Sialic acid, Influenza virus

## Abstract

**Background:**

Otitis media (OM) affects ≥80% of children before the age of three. OM can arise following co-infection with influenza A virus (IAV) and the bacterium *Streptococcus pneumoniae*. We have previously shown that H3 IAV strains (such as Udorn/72) induced a higher rate of bacterial OM than H1 strains (such as PR8/34). This was associated with more efficient replication of H3 strains in the middle ear.

**Findings:**

Here, we assess if the increased replication of IAV strains such as Udorn/72 in the middle ear is dependent upon the binding of the viral HA to α2,6-linked sialic acid. Using murine and *in vitro* models, the present study shows that recognition of α2,6-linked sialic acid was not required to facilitate bacterial OM.

**Conclusions:**

Taken together, these data suggest that other features of the HA mediate bacterial OM.

## Findings

### Introduction

Otitis media (OM) is the most frequently diagnosed illness in children under the age of five and can lead to permanent hearing loss [1]. OM is a polymicrobial disease and can arise following co-infection with influenza A virus (IAV) and the bacterium *Streptococcus pneumoniae* (the pneumococcus) [1]. We and others have demonstrated that infection with IAV facilitates the development of pneumococcal OM [2-4]. Specifically, we showed that H3 IAV strains, such as Udorn/72 (H3N2), induced a higher rate of pneumococcal OM in mice compared to H1 strains, such as PR8/34 (H1N1) [5]. This was related to the ability of the H3 hemagglutinin (HA) to mediate increased viral replication in the middle ear. H3 viruses also infected human middle ear epithelial cells (HMEECs) at a higher rate than H1 strains *in vitro*[5]. Here, we investigate if the increased replication of H3 strains in the middle ear is related to the sialic acid preference of the HA.

The HA of IAV binds to terminal sialic acid residues on cell surface glycoproteins and glycolipids, facilitating virus internalisation [6]. Human influenza viruses typically display a preference for sialic acid residues linked to cell surface glycans by an α2-6 linkage [6]. In humans, α2-6 linkages are most prevalent in the upper respiratory tract, whilst both α2-3 and α2-6 linkages are found in the lower respiratory tract [7]. Interestingly, whilst pre-1977 H3N2 strains like Udorn/72 recognise both α2,3- and α2,6-linked sialic acid, later H3N2 strains have evolved to recognise only α2,6-linked sialic acid [8,9]. In contrast to humans, α2,3-linked sialic acid dominates the murine respiratory tract [10]. Accordingly, virus strains adapted for murine infections (such as PR8/34) preferentially recognise α2,3-linked sialic acid [9]. Given that we have previously demonstrated that the Udorn/72 HA mediates efficient viral replication in the middle ear (and thus triggers bacterial OM) whilst the PR8/34 HA does not [5], the recognition of α2,6-linked sialic acid may facilitate increased viral replication in the middle ear. However, the sialic acid linkages present in the middle ears of infant C57BL/6 mice has, to the best of our knowledge, yet to be described. Here, we use a mouse model of OM and *in vitro* cultured HMEECs to show that increased replication of select H3 influenza virus strains in the middle ear is independent of tropism for α2,6-linked sialic acid.

## Materials & methods

### Viral and bacterial strains

The bioluminescent *S. pneumoniae* strain EF3030^lux^ (type 19F) was used in all experiments [11]. A/Udorn/307/72 (H3N2; Udorn72), A/Port Chalmers/1/73 (H3N2; Port Chalmers/73) and HKx31 (H3N2) were grown in embryonated eggs [4]. Horse-serum resistant (HS^R^) mutants were selected by growing the parent virus in embryonated eggs in the presence of non-immune horse serum. Tropism of all HS^R^ strains for only α2,3-linked sialic acid was confirmed by the desialyation-resialyation of red blood cells [9], and was consistent with previous publications with these strains [9].

### Infection of mice

Animal experiments were approved by the Animal Ethics Committee of The University of Melbourne (APP0811053 & APP1011872). C57BL/6 mice were bred and housed under specific pathogen-free conditions. Five-day old C57BL/6 mice were colonised intranasally (i.n.) with 2 × 10^3^ colony forming units (CFU) of *S. pneumoniae* EF3030^Lux^ or PBS in 3 μLs. At 14-days of age, mice were infected i.n. with 20 (PR8/34) or 10^2.5^ (all other virus strains) plaque forming units (PFU) of IAV in 3 μLs. Six days post-IAV infection, organs were collected for analysis.

### Enumeration of bacterial and viral load

Tissues used to quantify bacterial and viral load were collected and processed as described previously [11].

### Infection and staining of HMEECs

HMEECs [12] were kindly donated by Prof. David Lim (House Ear Institute, CA) and were infected and stained as described previously [5]. The percentage of infected cells was calculated by counting a minimum of 80 cells per well, where each data point was the average of an experimental duplicate.

### Lectin staining

Middle ears were collected and sectioned for histology essentially as described previously [4]. Lectin staining on middle ear sections was performed using the DIG glycan differentiation kit (Roche Diagnostics, Germany). Briefly, sections were incubated for 1 hour at 37°C in PBS or PBS supplemented with 400 mU/mL *Vibrio cholerae* sialidase (Sigma, USA). Sections were subsequently labelled with DIG-labelled *Maackia amurensis* agglutinin (MAA; α2,3-linked sialic acid) or *Sambucus nigra* agglutinin (SNA; α2,6-linked sialic acid) and stained with anti-DIG-FITC (Roche, USA). Autofluorescence was quenched using 0.01% (w/v) Evans blue (Sigma).

HMEECs were incubated with PBS supplemented with 4 mM CaCl_2_ ± 500 mU/mL *Vibrio cholerae* sialidase (Sigma) for 120 minutes at 37 degrees. Cells were subsequently washed and stained with biotinylated *Maackia amurensis* Lectin (MAL II) (Vector Laboratories, USA), or with biotinylated *Sambucus nigra* lectin (SNA I) (EY Laboratories, USA). Cells were once again washed, incubated with streptavidin-conjugated APC (BD Biosciences, USA) and lectin binding was measured on a LSR II flow cytometer (BD Biosciences, USA). Data was analysed with FlowJo version 7.6.5 (TreeStar Inc, Ashland, OR).

## Results & discussion

### OM is not associated with tropism of the viral HA for α2,6-linked sialic acid

**Figure 1:**
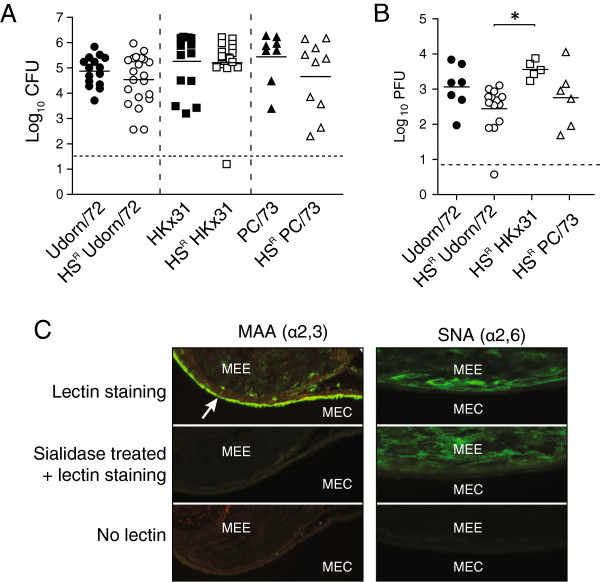
**α2,6-linked sialic acid recognition does not affect the development of pneumococcal OM. A**. Titres of *S. pneumoniae* EF3030^lux^ in the middle ears of mice six-days after i.n. infection with wild-type or horse-serum resistant (HS^R^) IAV strains. Bacterial counts are represented as the average titre derived from the left and right ear of each mouse. Data is pooled from a minimum of two independent experiments. Statistical significance was determined using a Mann–Whitney *U*-Test between the relevant wild-type or HS^R^ strain. Dashed line indicates the detection limit of the assay. **B**. Titres of IAV in the middle ears of mice six-days after i.n. infection with wild-type or horse-serum resistant (HS^R^) IAV strains. Viral tires are represented as the average titre derived from the left and right ear of each mouse. Data is pooled from a minimum of two independent experiments. Statistical significance was determined using a One-Way ANOVA with a Bonferroni post-correction and is denoted by an asterisk (p < 0.05). Dashed line indicates the detection limit of the assay. **C**. Sialic acid expression in the murine middle ear. Sections were labeled with *Maackia amurensis* agglutinin (MAA; α2,3-linked sialic acid) or *Sambucus nigra* agglutinin (SNA; α2,6-linked sialic acid) with or without pre-treatment with a sialidase. Positive staining is shown by an arrow. MEE: Middle Ear Epithelium; MEC: Middle Ear Cavity.

To determine if recognition of α2,6-linked sialic acid was important in the development of secondary bacterial OM, we assessed the ability of a horse serum-resistant mutant (HS^R^) of Udorn/72 to cause pneumococcal OM. This virus has a single amino acid substitution at residue 226 of the HA that changes its specificity from dual recognition of α2,3- and α2,6-linked sialic acid, to α2,3-linked sialic acid only [9]. Mice colonised with *S. pneumoniae* at 5-days old were then infected with wild-type Udorn/72 or HS^R^ Udorn/72 at 14-days old. Pneumococcal OM was assessed by measuring bacterial titres in the middle ear six-days post-IAV infection. This time-point was selected as we have previously shown that six days after IAV infection is the peak of bacterial OM in infant mice [4]. Mice infected with HS^R^ Udorn/72 displayed no significant difference in middle ear bacterial titres compared to mice infected with wild-type Udorn/72 (Figure 1A; Mann–Whitney *U*-Test, p > 0.05). Accordingly, there was no difference in viral replication between wild-type and HS^R^ Udorn/72 in the middle ear six-days post IAV infection (Figure 1B; One-Way ANOVA with a Bonferroni post-correction, p > 0.05). Like Udorn/72, the H3N2 viruses HK×31 and PC/73 also replicated efficiently in the middle ear and facilitated bacterial OM [5]. As these viruses also display a dual-receptor specificity [9,13], we used HS^R^ mutants to assess if bacterial OM caused by HKx31 and PC/73 was also independent of tropism for α2,6-linked sialic acid. HS^R^ HKx31 and HS^R^ PC/73 did not mediate differential bacterial replication in the middle ear relative to their wild-type parental strains (Figure 1A; Mann–Whitney *U*-Test, p > 0.05). Similarly, there were no significant differences in the titres of these viruses in the middle ear relative to Udorn/72 (Figure 1B; One-Way ANOVA with a Bonferroni post-correction, p > 0.05) and their replication was equivalent to relevant parental strains [5]. These observations were supported by immunofluorescent staining of middle ears of uninfected mice, which demonstrated that this site is rich in α2,3-linked sialic acid (Figure 1C). In contrast, only non-specific background staining was observed for α2,6-linked sialic acid (Figure 1C). The middle ears of *S. pneumoniae*, IAV and co-infected mice were also rich in α2,3-linked sialic acid (Additional file 1). Furthermore, we have previously demonstrated that secondary pneumococcal OM is independent of the viral NA and that the pneumococci in the ear localise to the lumen rather than the middle ear epithelium [4,5]. Thus, α2,6-linked sialic acid recognition is not the critical determinant of virus replication in the middle ear or secondary bacterial OM.

### Tropism for α2,6-linked sialic acid does not determine the *in vitro* infection rate of human middle ear epithelial cells by Udorn/72

**Figure 2:**
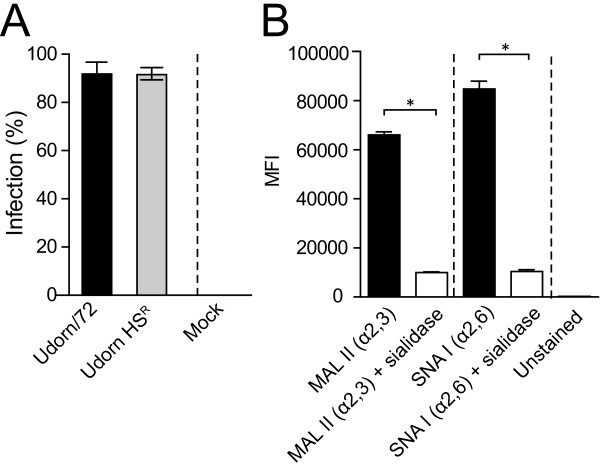
**α2,6-linked sialic acid recognition does not affect infection of human middle ear epithelial cells (HMEECs). A**. Percentage of HMEECs infected by wild-type Udorn/72 or horse serum resistant (HS^R^) Udorn/72 eight hours post-infection at an MOI of 1. Statistical significance was determined using a Mann–Whitney *U*-Test. Data represents mean ± SEM. **B**. HMEECs express α2,3- and α2,6-linked sialic acid. HMEECs were incubated with PBS containing 1% BSA (unstained), biotinylated MAL II (to detect α2,3-linked sialic acid), biotinylated SNA I (to detect α2,6-linked sialic acid) or biotinylated MAL II or SNA I following sialidase treatment (sialidase treated). Cells were subsequently stained with streptavidin-conjugated APC and measured by flow cytometry. The mean fluorescent intensity (MFI) is shown. Data is pooled from two independent experiments where each experiment was performed in duplicate. Data represents mean ± SEM. Statistical significance was determined using a Mann–Whitney *U*-Test and is denoted by one asterisk (p < 0.05).

To assess the relevance of these findings to human infections, HMEECs were infected with Udorn/72 or HS^R^ Udorn/72. HS^R^ Udorn/72 was not significantly reduced in its ability to infect HMEECs when compared to the wild-type Udorn/72 (Figure 2A; Mann–Whitney *U*-Test, p > 0.05). This suggests that recognition of α2,6-linked sialic acid on HMEECs by the Udorn/72 HA is not the major factor in determining the rate of infection. Consistent with this hypothesis, HMEECs expressed both α2,6- and α2,3-linked sialic acid on the cell surface (Figure 2B). The specificity of this binding was confirmed by the reduced number of fluorescent cells observed following sialidase treatment of HMEECs (Figure 2B).

In summary, we have previously demonstrated that H3 viruses (such as Udorn/72) with dual specificity for α2,6- and α2,3-linked sialic acid facilitate bacterial OM. In contrast, H1 viruses (such as PR8/34) which only recognise α2,3-linked sialic acid, do not facilitate bacterial OM. We therefore reasoned that recognition of α2,6-linked sialic acid may promote increased viral replication in the middle ear and thus bacterial OM. However, H3 viruses with a specificity for only α2,3-linked sialic acid were still effective at inducing bacterial OM, replicating in the middle ear and infecting HMEECs. At present, it remains unclear what alternative feature of the H3 HA mediates increased viral replication in the middle ear and thus bacterial OM. Following sialic acid-mediated viral attachment, the H3 HA may promote more effective interactions with secondary receptors required for virus entry and replication [14]. Alternatively, H3 HAs may display increased membrane fusion with epithelial cells in the middle ear, thereby promoting more efficient viral replication [15]. Thus, whilst OM is independent of tropism for α2,6-linked sialic acid, the other features of the HA which may mediate secondary bacterial disease remain an area of ongoing research.

## Abbreviations

CFU: Colony forming units; HA: Hemagglutinin; HSR: Horse serum resistant; HMEECs: Human middle ear epithelial cells; IAV: Influenza A virus; i.n: intranasally; OM: Otitis media; PFU: Plaque forming units.

## Competing interests

The authors declare that they have no competing interests.

## Authors’ contributions

KRS: Performed experiments, assisted in study design and wrote the manuscript; MNH & JP: Performed experiments, study design and data analysis; PCR/OLW: Assisted in study design & editing manuscript DAD: Assisted in study design and data analysis, performed experiments and helped draft manuscript. All authors read and approved the final manuscript.

## Supplementary Material

Additional file 1. Figure S1 Sialic acid expression in the murine middle ear following infection with *Streptococcus pneumoniae* (Sp) and/or the IAV strain Udorn/72. Sections were labeled with *Maackia amurensis* agglutinin (MAA; α2,3-linked sialic acid), *Sambucus nigra* agglutinin (SNA; α2,6-linked sialic acid) or unlabelled. Positive staining is shown by an arrow. MEE: Middle Ear Epithelium; MEC: Middle Ear Cavity. Click here for file
