# Supplementary figures and images for: Influenza A virus induced bacterial otitis media is independent of virus tropism for α2,6-linked sialic acid
Source: Virol J. 2013 Apr 23;10:128. doi: 10.1186/1743-422X-10-128 (PMC3655017; doi:10.1186/1743-422X-10-128)

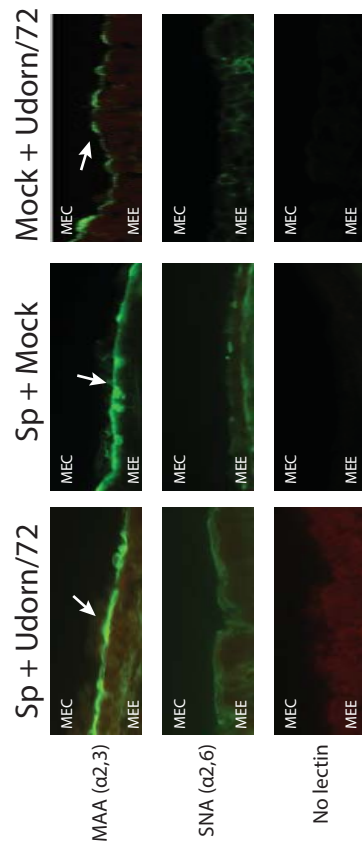

Supplement: Additional file 1. Figure S1 — Sialic acid expression in the murine middle ear following infection with Streptococcus pneumoniae (Sp) and/or the IAV strain Udorn/72. Sections were labeled with Maackia amurensis agglutinin (MAA; α2,3-linked sialic acid), Sambucus nigra agglutinin (SNA; α2,6-linked sialic acid) or unlabelled. Positive staining is shown by an arrow. MEE: Middle Ear Epithelium; MEC: Middle Ear Cavity. [file 1743-422X-10-128-S1.pdf]
